# Expression of Concern: The RNA-binding protein CUG-BP1 increases survivin expression in oesophageal cancer cells through enhanced mRNA stability

**DOI:** 10.1042/BCJ20120112_EOC

**Published:** 2026-07-24

**Authors:** Elizabeth T. Chang, James M. Donahue, Lan Xiao, Yuhong Cui, Jaladanki N. Rao, Douglas J. Turner, William S. Twaddell, Jian-Ying Wang, Richard J. Battafarano

**Affiliations:** 1 Cell Biology Group, Department of Surgery, University of Maryland School of Medicine, Baltimore, MD 21201, U.S.A.; 2 Baltimore Veterans Affairs Medical Center, Baltimore, MD 21201, U.S.A; 3 Department of Pathology, University of Maryland School of Medicine, Baltimore, MD 21201, U.S.A.

**Keywords:** 3′-untranslated region, apoptosis, CUG-binding protein 1 (CUG-BP1), mRNA stability, post-transcriptional regulation, survivin

The *Biochemical Journal* has been made aware of potential issues surrounding the scientific validity of this paper and is hence issuing a permanent Expression of Concern to notify readers.

A reader notified us of similarities between the Figure 1B second and third Actin bands and the Figure 3E TE7 and TE10 Actin bands. Upon further investigation, the Editorial Office has noted similarities between the C-siRNA 400/GAPDH bands of Figure 3F and 3G. We have been unable to get in touch with the corresponding author, and whilst we received a response from one of the co-authors of the article, they were unable to provide any raw data or explanations into the similarities.

The Editorial Board has therefore decided to issue a permanent Expression of Concern for this article to alert readers to our concerns.

